# Noninvasive Electrical Modalities to Alleviate Respiratory Deficits Following Spinal Cord Injury

**DOI:** 10.3390/life14121657

**Published:** 2024-12-13

**Authors:** Niraj Singh Tharu, Aastha Suthar, Yury Gerasimenko, Camilo Castillo, Alex Ng, Alexander Ovechkin

**Affiliations:** 1 Kentucky Spinal Cord Injury Research Center, University of Louisville, Louisville, KY 40202, USA; niraj.tharu@louisville.edu (N.S.T.); aastha.suthar@louisville.edu (A.S.); 2 Department of Neurological Surgery, University of Louisville, Louisville, KY 40202, USA; 3 Department of Physiology, University of Louisville, Louisville, KY 40202, USA; yury.gerasimenko@louisville.edu; 4 Pavlov Institute of Physiology Russian Academy of Sciences, St. Petersburg 199034, Russia; 5 Department of Neurological Surgery, Division of Physical Medicine and Rehabilitation, University of Louisville, Louisville, KY 40202, USA; camilo.castillo@uoflhealth.org; 6 Department of Medicine, Division of Pulmonary, Critical Care, and Sleep Disorders Medicine, University of Louisville, Louisville, KY 40202, USA; alex.ng@louisville.edu

**Keywords:** transcutaneous spinal cord stimulation, noninvasive electrical stimulation, respiration, respiratory motor control, cough, spinal cord injury

## Abstract

(1) Background: Respiratory dysfunction is a debilitating consequence of cervical and thoracic spinal cord injury (SCI), resulting from the loss of cortico-spinal drive to respiratory motor networks. This impairment affects both central and peripheral nervous systems, disrupting motor control and muscle innervation, which is essential for effective breathing. These deficits significantly impact the health and quality of life of individuals with SCI. Noninvasive stimulation techniques targeting these networks have emerged as a promising strategy to restore respiratory function. This study systematically reviewed the evidence on noninvasive electrical stimulation modalities targeting respiratory motor networks, complemented by previously unpublished data from our research. (2) Methods: A systematic search of five databases (PubMed, Ovid, Embase, Science Direct, and Web of Science) identified studies published through 31 August 2024. A total of 19 studies involving 194 participants with SCI were included. Unpublished data from our research were also analyzed to provide supplementary insights. (3) Results: Among the stimulation modalities reviewed, spinal cord transcutaneous stimulation (scTS) emerged as a particularly promising therapeutic approach for respiratory rehabilitation in individuals with SCI. An exploratory clinical trial conducted by the authors confirmed the effectiveness of scTS in enhancing respiratory motor performance using a bipolar, 5 kHz-modulated, and 1 ms pulse width modality. However, the heterogeneity in SCI populations and stimulation protocols across studies underscores the need for further standardization and individualized optimization to enhance clinical outcomes. (4) Conclusions: Developing standardized and individualized neuromodulatory protocols, addressing both central and peripheral nervous system impairments, is critical to optimizing respiratory recovery and advancing clinical implementation.

## 1. Background

Spinal cord injury (SCI) leads to significant impairment or loss of respiratory motor control, with severe consequences that profoundly affect overall health and quality of life [1,2,3,4]. The disruption of respiratory motor function is closely linked to the level and completeness of the neurological injury, with cervical and upper thoracic injuries posing the greatest risk of severe respiratory dysfunction [4,5]. Individuals with these injuries often experience reduced respiratory rates, diminished breathing capacity, impaired airway clearance, and compromised coughing ability, all of which interfere with daily activities and increase morbidity [6,7]. Restoring respiratory function in these patients is critical to improving health outcomes and quality of life [8,9].

Respiratory complications are a significant concern following SCI, as they represent one of the leading causes of morbidity and mortality in this population [6,10,11,12]. These complications often emerge immediately after the injury and can persist or evolve over time. In the acute phase (days to weeks post-injury), individuals frequently experience respiratory muscle weakness or paralysis, impaired airway clearance, and reduced lung compliance due to neural disruption at or above the cervical spinal cord [13]. This phase is characterized by an increased risk of atelectasis, pneumonia, and respiratory failure, necessitating intensive medical support. Over the subacute (weeks to months) and chronic phases (months to years post-injury), respiratory dysfunction often manifests as reduced vital capacity, chronic hypoventilation, and ineffective coughing, further predisposing individuals to recurrent infections and diminished quality of life [13,14,15].

The timing and severity of these complications vary depending on the level and completeness of the SCI. Injuries at the cervical level, particularly those involving segments C3–C5, are associated with significant impairment of the phrenic nerve, which innervates the diaphragm, the primary muscle of respiration [13,16]. Upper thoracic injuries also compromise accessory respiratory muscles, such as the intercostals and abdominal muscles, exacerbating respiratory inefficiencies. Without targeted interventions, these complications often become chronic, highlighting the urgent need for therapeutic approaches that can support respiratory function throughout the continuum of recovery [17,18].

Despite advancements in therapy over the years, SCI remains incurable [2]. Traditional approaches have focused primarily on functional mobility, with less emphasis on targeted respiratory interventions [19,20,21]. To address these gaps, electrical stimulation has emerged as a promising strategy for enhancing impaired functions, including respiratory recovery [22,23]. This review focuses on noninvasive modalities such as functional electrical stimulation (FES), neuromuscular electrical stimulation (NMES), functional magnetic stimulation (FMS), and spinal cord transcutaneous stimulation (scTS), which aim to improve respiratory function and cough.

### 1.1. Evolution of Electrical Stimulation in SCI Rehabilitation

The therapeutic application of electrical stimulation dates back to ancient Rome, where electric shocks from ray fish were used to relieve pain [22,23]. Modern electrotherapy was introduced in the 1960s and 1970s as a method to alleviate pain [24], and its applications gradually expanded to physical rehabilitation for neurological disorders [25]. Electrical stimulation is now widely used in SCI to augment neural signaling and potentially restore impaired functions [24].

FES and NMES are two commonly applied modalities in SCI rehabilitation. FES activates paralyzed muscles to restore functional activity, while NMES stimulates peripheral motor nerves to strengthen muscles or improve mobility [26]. FES, first used in 1961 to address foot drop after stroke [27], has since been applied to SCI, showing promise in enhancing functional activity and respiratory outcomes [22]. However, its efficacy depends on combining electrical stimulation-induced activity with voluntary effort, as passive stimulation alone may not be sufficient [27,28]. Devices such as EMPI (St. Paul, Minnesota, USA), DS7 (Digitimer Co, Hertfordshire, UK), and Rehastim v1 (Hasomed, Germany) have demonstrated improvements in cough strength and expiratory flow, likely through enhanced abdominal muscle activation [29,30,31,32,33,34]. This improvement may be attributed to enhanced abdominal muscle mass and tone, which, in turn, improve respiratory performance [35].

NMES, first applied in 1964 for stroke rehabilitation [36], has been used in SCI to improve respiratory function by activating abdominal muscles important for breathing and coughing [37]. While NMES shows benefits in re-educating muscles and reducing spasticity, it has limitations such as discomfort and inadequate muscle fiber recruitment [38,39,40]. Unfortunately, for electrical stimulation modalities, the number of terminologies has developed uncertainty regarding the physiologic effects and clinical results [39]. Studies with devices like Phrenics Dualpex Quark^®^ and EMPI have demonstrated improvements in inspiratory and expiratory pressures, suggesting enhanced coughing and secretion clearance [37,41]. The maximum inspiratory and expiratory pressure were improved with increased peak expiratory flow, indicating enhanced coughing ability, which assisted in clearing bronchial secretions. It was believed that muscle contractions caused by NMES developed intrathoracic pressure swing during cough, thus removing the discharge from peripheral airways [37].

FMS has also been explored for respiratory recovery. First demonstrated in 1896, FMS was later applied in 1989 to stimulate the phrenic nerve, potentially supporting thoracic neural network activation in SCI [42]. Despite promising results, such as increased expiratory flow and thoracic nerve activation, FMS remains underutilized and largely confined to laboratory settings [28].

### 1.2. Emergence of scTS for Respiratory Recovery

Beginning in the 1960s, spinal cord stimulation emerged as an invasive epidural therapy for the treatment of chronic pain [43]. In 1967, Dr. Norman Shealy implanted subdural electrodes at the dorsal columns of T2–T3 to alleviate pain in a patient with inoperable bronchogenic cancer [44]. This groundbreaking work paved the way for Shealy and colleagues to establish the therapeutic efficacy of epidural stimulation for managing neuropathic pain [45]. While early research primarily focused on invasive epidural stimulation, the introduction of scTS in the late 1990s demonstrated the feasibility of accessing low-threshold sites on the human lumbosacral cord to induce muscular twitches noninvasively. A decade later, studies extensively investigated and characterized the motor reflexes elicited by scTS, confirming its ability to activate posterior root afferents [43].

Recently, scTS has emerged as a promising therapy for restoring and enhancing impaired functions after SCI. This technique stimulates spinal circuitries and activates muscles innervated by the targeted spinal segments, with stimulation electrodes placed over the spinal cord. Its noninvasive nature, user-friendly application, and cost-effectiveness make scTS an appealing alternative to epidural stimulation [43,46]. However, the clinical adoption of scTS is hindered by a lack of robust scientific evidence [23]. Challenges include the absence of standardized stimulation parameters and limited understanding of the underlying mechanisms [47]. Recent studies on scTS have shown promise for respiratory recovery. Devices like BioStim-5 (Cosyma Inc., Denver, CO, USA) and TESCoN (spineX, Inc., USA) have demonstrated increased inspiratory capacity and enhanced electromyographic activity in the diaphragm [48]. scTS offers additional advantages for individuals unable to undergo surgical interventions, as it can activate broader spinal networks and target multiple organ systems through simple adjustments in electrode positioning [19,48]. However, challenges such as inconsistent stimulation parameters, a limited understanding of underlying mechanisms, and risks like autonomic dysreflexia and skin injury hinder its widespread adoption [23,30,47].

Our previous work demonstrated that respiratory functional outcomes can be significantly improved using our original reciprocal inspiratory–/ expiratory pressure threshold respiratory training (RT) protocol [49]. These improvements were associated with plasticity in respiratory motor and autonomic activity [50]. However, the effectiveness of this intervention is limited by the low excitability of spinal networks below the injury, restricting its impact to the functional capacity preserved post-injury [51]. Our group has pioneered the use of spinal cord epidural stimulation (scES) to activate motor [52] and autonomic [53] spinal networks in individuals with severe SCI. In studies investigating trunk and lower limb muscle activation during voluntary tasks in a patient with cervical motor-complete SCI (C7 AIS-B), we observed that respiratory muscles below the injury level could be activated when these tasks were assisted by scES applied below the lesion [54]. Additionally, our research demonstrates that tonic spinal cord stimulation below the lesion induces voltage-dependent changes in breathing patterns and enhances voluntary respiratory muscle activation below the injury level [55]. The data presented here strongly suggest that scTS can further enhance adaptive plasticity and amplify therapeutic effects when combined with RT.

### 1.3. Research Gaps and Study Objectives

Although electrical stimulation modalities have shown potential for respiratory recovery in SCI, existing studies use diverse protocols and parameters, resulting in varied outcomes and inconsistent evidence [8,9]. A significant number of studies fail to adequately document stimulation configurations, further complicating efforts to establish standard protocols [9,20]. Addressing these gaps is critical to developing effective, replicable interventions tailored to individual needs [22,56,57].

This study aims to (i) identify and detail the specific stimulation parameters and protocols of noninvasive electrical stimulation modalities that show promise in enhancing respiratory function and cough, (ii) conduct a pilot efficacy trial to evaluate the application of optimal scTS parameters, derived from the literature, in activating respiratory motor networks in individuals with SCI, and (iii) provide actionable insights for researchers and clinicians on implementing effective stimulation protocols to optimize outcomes in respiratory rehabilitation.

By addressing these objectives, this study aims to contribute to the development of standardized and evidence-based protocols for respiratory recovery.

## 2. Materials and Methods

### 2.1. Search Strategy

To ensure broad inclusion, the authors initially sought any publication related to noninvasive electrical stimulation modalities targeting autonomic functions, irrespective of the publication title. A comprehensive literature review was conducted, focusing on the stimulation parameters of these modalities. Systematic searches were performed across five electronic databases, namely PubMed, Ovid, Embase, Science Direct, and Web of Science, to identify studies published in English. Additionally, manual searches were conducted using Google Scholar to retrieve relevant publications, clinical trials, and systematic reviews. Bibliographies of these sources were further screened to identify additional eligible studies. The primary focus of the search was on the restoration of autonomic functions, particularly respiratory function and cough, following SCI. Given the limited number of eligible studies, no restrictions were placed on the publication year or sample size. The literature search was last updated on 31 August 2024.

### 2.2. Search Terms

The following search terms were used: (i) participants: spinal cord injury, spinal cord injuries, cervical cord injury, thoracic cord injury, tetraplegia, quadriplegia, and paralysis. (ii) Interventions: electrical stimulation, functional electrical stimulation, neuromuscular electrical stimulation, functional magnetic stimulation, spinal cord transcutaneous stimulation, neuromodulation, and spinal cord stimulation. (iii) Outcomes: autonomic functions, cough, breathing, respiratory function, respiratory recovery, pulmonary function.

### 2.3. Search Screening and Selection

**Figure 1:**
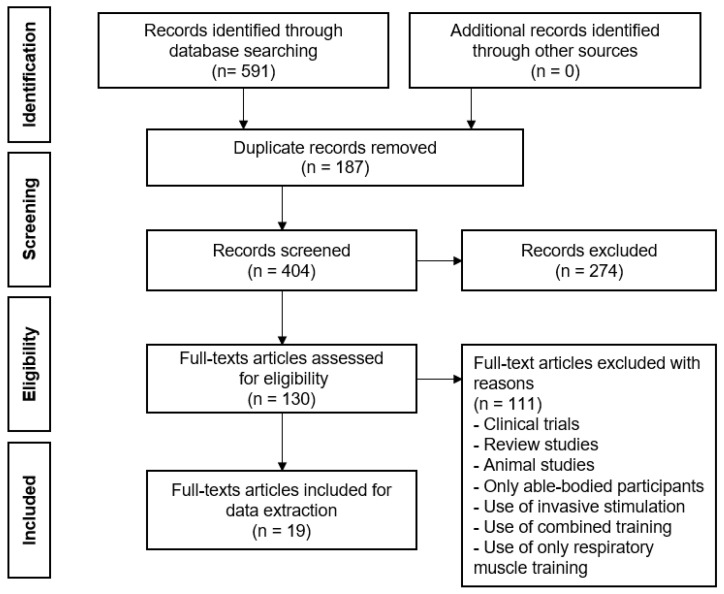
A flow diagram representing the article identification, screening, review, and selection process.

Following an initial screening of study titles and abstracts, full-text screening was carried out once the articles were retrieved. The chosen articles were transferred to EndNote version 20.2.1 and duplicate articles were removed. Furthermore, eligibility was determined through reviewing the full-text versions of every relevant study (Figure 1). In addition, the Modified Downs and Black checklist was used to assess the risk of bias, and three reviewers assessed the selected studies independently.

### 2.4. Search Inclusion and Exclusion Criteria

The following inclusion criteria were applied: (i) participants: above the age of 18 years who had incomplete or complete SCI with at any times post-SCI. (ii) Interventions: studies using noninvasive electrical stimulation modalities along with respiratory muscle training and/or any respiratory training. (iii) Outcomes: studies showing improvement in at least one measure of pulmonary functions, i.e., breathing and cough. Similarly, the studies excluded had participants with conditions other than SCI, one stimulation modality paired with another stimulation modality, studies that failed to specify stimulation parameters and protocols, with abstracts not available in the English language, animal studies, and studies with outcomes that measured anything other than respiratory function and cough.

### 2.5. Risk of Bias Assessment

Three independent reviewers conducted these assessments to ensure reliability and minimize subjective bias. Studies that demonstrated significant methodological weaknesses, such as inadequate reporting of outcomes, lack of participant details, or incomplete descriptions of stimulation protocols, were excluded from the analysis. Furthermore, studies with unclear or inconsistent methodologies that could compromise the validity of their findings were also excluded. The results of the risk of bias assessments were not used to arbitrarily exclude studies but rather to prioritize the inclusion of high-quality evidence that met the predefined eligibility criteria.

### 2.6. Search Data Extraction and Synthesis

The purpose of this study was to identify appropriate stimulation parameters and protocols which produced the greatest outcomes through extracting data from each selected article about their stimulation configurations. The included publications were reviewed independently, and the data that were retrieved contained details about the participants, including their mean age, gender, the number of subjects, length of injury, severity of damage, and American Spinal Cord Injury Association Impairment Scale (AIS) classification. Furthermore, data concerning the intervention included experimental details, the stimulation region, the duration of the intervention, characteristics of the stimulation, outcome measures, the effect of treatment, and adverse effects. The extracted data were presented in the form of tables and figures. The electrical stimulation modalities included in this paper used a diverse set of stimulation protocols and parameters, making it difficult to compare. There is also inconsistency in the way these parameters are explained.

### 2.7. Exploratory Trial Procedures

The study was approved by the University of Louisville Institutional Review Board (IRB # 23.0570). Two female participants both diagnosed with C4 AIS-B SCI, aged 47 ± 3 years, were recruited 56 ± 12 mos. after injury. Throughout the study, participants maintained their regular daily activities, and none withdrew from the study.

Datasets were collected in a physiological laboratory environment during 2 visits for each participant using test–re-test methodology. During the interventional sessions, participants were seated in a chair with an approximately 45° head-up tilt. Multi-site scTS was applied using a Biostim-5 device (Cosyma Inc., Denver, CO, USA). Stimulation was delivered as 5 kHz-modulated monophasic pulses with a duration of 1 ms at a frequency of 30 Hz, targeting dorsal root activation.

Self-adhesive circular electrodes (32 mm diameter, ValueTrode, Axelgaard Manufacturing Co., Ltd., Fallbrook, CA, USA) were placed midline over the thoracic spine at the levels corresponding to the T5 and T8 spinal cord segments (Th3-Th4 and Th8-Th9 spinous processes). Two rectangular electrodes (5 × 9 cm, ValueTrode, Axelgaard Manufacturing Co., Ltd., Fallbrook, CA, USA) were positioned bilaterally along the rectus abdominis muscles at the umbilical level, serving as anodes [58]. The stimulation intensity was incrementally increased, starting at 10 mA, until the motor threshold was reached, identified by visible twitching of the intercostal or abdominal muscles. Average thresholds were 42.3 ± 10.7 mA at the Th3-Th4 site and 40.1 ± 11.6 mA at the Th8-Th9 site. During the intervention phase, stimulation intensity was reduced by 10 mA to remain below the motor threshold. Physiological parameters, including brachial arterial blood pressure (BP) and heart rate (HR), were monitored before stimulation (20 min), during stimulation, and for 20 min post-intervention using Caretaker System VER 8.0 (Caretaker Medical Corp., Charlottesville, VA, USA).

### 2.8. Outcome Measures

The acute effects of scTS were evaluated by measuring maximum expiratory airway pressure (PEmax) and surface electromyography (sEMG) values before and during the scTS exposure. Six PEmax attempts were performed before and during scTS during each visit (2 visits, 24 measurements total). PEmax measures were recorded using an MP45-36-871-350 Differential Pressure Transducer (Validyne Engineering, Northridge, CA, USA), and sEMG was captured with an MA300-XVI EMG system (Motion Lab Systems, Lake Elsinore, CA, USA). sEMG signals were obtained from the left and right clavicular portions of the pectoralis (PEC, on the midclavicular line), intercostal (IC, at the 6th intercostal space on the anterior axillary line), rectus abdominis (RA, at the umbilical level), and obliquus abdominis (OBL, on the midaxillary line) muscles.

### 2.9. Statistical Analysis

Outcomes that were normally distributed, as determined by the Shapiro–Wilk test, were summarized using the mean and standard deviation (SD) and evaluated with a paired two-sided t-test. Pre-post changes were quantified using “Cohen’s d” effect size [59], calculated as the mean of the differences divided by the SD of differences for normally distributed outcomes. Effect sizes were classified according to Sawilowsky’s extension [60] of Cohen’s criteria as trivial (<0.01), very small (0.01–0.19), small (0.2–0.49), medium (0.5–0.79), large (0.8–1.19), very large (1.2–1.99), and huge (≥2.0). A threshold of 0.5 was considered the minimally important difference for health-related quality of life [61,62]. The statistical significance threshold was set to α = 0.05.

## 3. Results

### 3.1. Articles Retrieved

The articles were systematically retrieved from five databases (PubMed, Ovid, Embase, Science Direct, and Web of Science). A total of 591 records were found and transferred to EndNote to eliminate any duplicate entries. The title and abstract of 404 records were carefully screened, resulting in the exclusion of 274 records. In addition, the remaining 130 full-text articles were evaluated to determine their eligibility, resulting in the elimination of 111 articles that did not match the requirements for inclusion. Finally, a total of 19 (FES = 14, NMES = 2, FMS = 1, scTS = 2) articles were selected for data extraction (Figure 1).

### 3.2. Participant Characteristics

**Figure 2:**
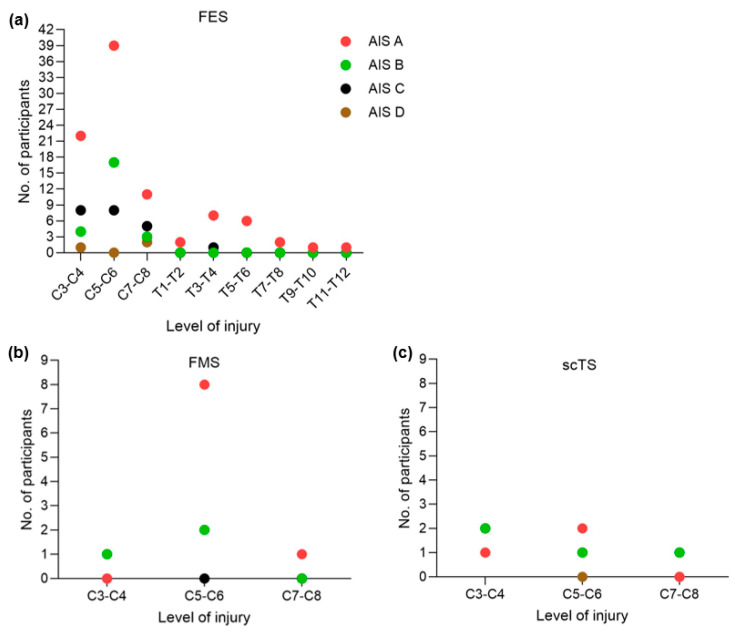
Participant characteristics presenting the level of injury and AIS classification for given noninvasive electrical stimulation modalities. (**a**) FES, (**b**) FMS, and (**c**) scTS. Abbreviations: FES = functional electrical stimulation; FMS = functional magnetic stimulation; scTS = spinal cord transcutaneous stimulation; AIS = American Spinal Cord Injury Association Impairment Scale.

Out of the 19 selected studies that were included in this analysis, only three (15.7%) were carried out within the last five years. About 170 males and 24 females with SCI were included in the studies that specified gender information. Among the 194 individuals, these treatments were administered to FES (M = 132; F = 20), NMES (M = 14; F = 3), FMS (M = 13; F = 0), and scTS (M = 11; F = 1). Eleven of the studies included recruited both sexes, while eight of the 19 studies used only male subjects. Men were the only subjects in six of the fourteen FES studies. In addition, only male participants were included in one scTS and FMS study. The mean age and time since the injury of participants with chronic SCI ranged between 34.1 and 46.9 years and 2.4–14.6 years, respectively. Out of the 19 studies that were considered, 13 of them only included participants with cervical SCI, whereas the remaining six included both cervical or thoracic SCI. The level of injury ranged between C3 and T12, where C5–C6 occupied the highest proportion, followed by C3–C4. Although 13 out of 19 studies reported AIS grades, six studies only used the terms “complete” and “incomplete” to describe the SCIs rather than AIS grades. The maximum number of study participants had their injury graded as AIS A followed by AIS B. The AIS classifications for FES, NMES, FMS, and scTS are described in Figure 2 and Table 1.

### 3.3. Stimulation Characteristics

Data in this study demonstrated that stimulation (FES, NMES, FMS, and scTS) was administered across an extensive region. FES was primarily delivered to abdominal, rectus abdominis, external oblique muscles, and posterolateral and thoracoabdominal walls. NMES targeted the sixth, seventh, and eighth intercostal spaces and the clavicular portion of the pectoralis major and abdominal muscles. It could be noticed that abdominal muscles were the commonly stimulated sites used for FES and NMES. The spinous process line is specifically stimulated through FMS and scTS. FMS was delivered between the T10–T11 spinous process, whereas scTS has been reported to stimulate C3–C4, C5–C6 and T1–T2, and T9–T10 regions. In addition, the majority of studies also failed to describe electrode sizes and anode or cathode electrode placements. Overall, these studies exhibited a wide range of electrode configurations with respect to placement and location.

The described stimulation techniques had an intervention duration ranging from weeks to months. Eight of the fourteen FES studies did not report the length of intervention, while the remaining six studies had their delivery of intervention ranging between 2 and 8 weeks and 30–60 min per session for about 5 to 7 days a week, reporting positive effects. One NMES study reported that the frequency, length, and time to deliver the stimulation was 30 min every day for 4 weeks, whereas another NMES study reported 7 days per week for 20 min but did not specify the duration it had been delivered. Again, the FMS study did not describe these parameters. Similarly, scTS studies delivered the intervention for 1–2 weeks for 5 days a week and 30–60 min daily (Table 2). Most studies showed that the length and sessions of stimulation delivery was observed to be 4–6 weeks for 5–7 days weekly, followed by 30 min each session. These findings indicate that the stimulation characteristics varied among reviewed studies, making it hard to draw definitive conclusions about the optimal stimulation duration, time, length, and number of sessions daily/weekly.

### 3.4. Stimulation Protocols

**Figure 3:**
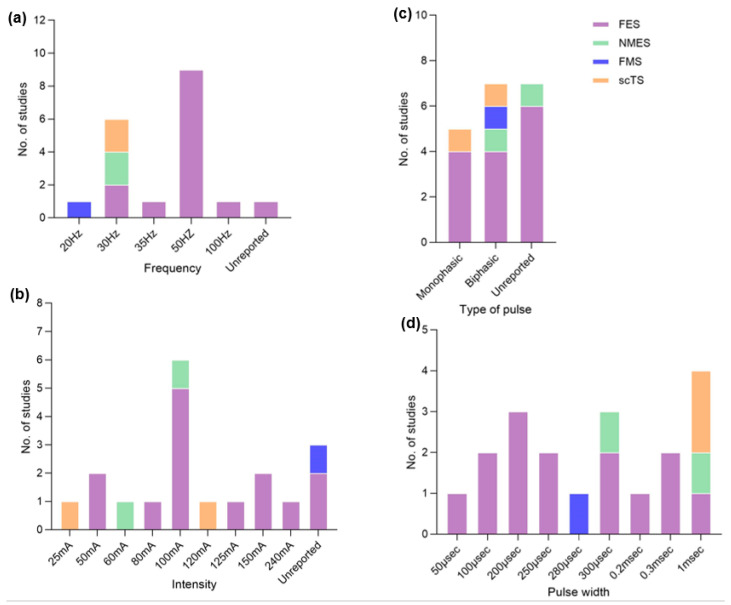
Stimulation parameters for given noninvasive electrical stimulation modalities. (**a**) Frequency; (**b**) intensity; (**c**) type of pulse; (**d**) pulse width. Abbreviation: FES = functional electrical stimulation; NMES = neuromuscular electrical stimulation; FMS = functional magnetic stimulation; scTS = spinal cord transcutaneous stimulation.

Figure 3 and Table 3 showed that for 7 out of 14 FES studies, the frequency of stimulation used was 50 Hz, with a pulse width between 0.3 and 1 ms, and delivered monophasic pulses where intensity ranged between 100 and 125 mA, eliciting strong muscle contraction. Furthermore, the remaining studies included monophasic or biphasic pulses with pulse duration ranging from 200 to 300 µs and frequencies ranging from 35 to 100 Hz, with the intensity being gradually increased till visible muscle contraction. NMES applied a frequency of 30 Hz with a pulse width ranging from 300 µs to 1 ms, and the current intensity ranged between 60 and 100 mA. For FMS, 20 Hz of frequency was utilized with a biphasic waveform of 280 µs pulse width, and stimulation intensity was not quantified. The scTS used monophasic or biphasic rectangular pulses with a pulse width of 1 ms and a frequency of 30 Hz, intensities ranging from 25 to 120 mA, and a carrier frequency of 10 kHz. Nine of fourteen FES studies reported the use of a frequency of 50 Hz, while another two studies used a frequency of 30 Hz. In addition, two studies reported the application of frequencies of 35 Hz and 100 Hz, respectively, followed by another study which did not specify the frequency used, and pulse width and current intensity varied across each study. FMS used a frequency of 20 Hz, while the other stimulation modalities (NMES and scTS) reported using a consistent frequency (30 Hz), but other stimulation parameters (pulse width, type of pulse, and intensity) varied. The extracted data showed that compared to the other stimulation modalities (FES, NMES, and scTS), FMS used the lowest frequency (20 Hz), while FES applied the higher frequency (100 Hz) and highest intensity (240 mA). Most of the included studies reported the use of a rectangular biphasic current waveform with varying pulse width, with five studies using monophasic pulses. Because stimulation protocols varied across various noninvasive electrical modalities, further research is warranted to establish standardization of the stimulation protocols. It is unreasonable to compare the stimulation characteristics and protocols among included studies due to the variations in electrode sizes and stimulation configurations, as well as variability in dosage parameters including amplitude, frequency, and pulse duration. Consequently, we tried to identify common stimulation characteristics and protocols derived from the data collected in this study.

### 3.5. Primary Outcome Measures

Of the 19 reviewed studies, 10 studies used forced vital capacity (FVC) and forced expiratory volume in 1 s (FEV1), seven studies applied the peak expiratory flow rate (PEFR), and the remaining two studies utilized tidal volume as their primary outcome measures to assess respiratory functions and cough. Out of 14 FES studies, three studies measured vital capacity (VC), FVC, FEV1, and PEFR to augment and assist coughing after SCI. Two studies calculated gastric and esophageal pressure; another two studies measured tidal volume (VT) and PEFR to observe the effects of ES to produce cough. In addition, five FES studies focused on studying changes in pulmonary function measures and enhancing cough, where they mainly assessed maximum expiratory pressure (MEP) and maximum inspiratory pressure (MIP), PEFR, maximum expiratory cough pressure (MECP), FVC, and FEV1. Furthermore, one study measured the level of oxygen intake (VO2) and rate of perceived exertion (RPE), while another study calculated gastric and esophageal pressure to investigate the effect of FES on cough and oxygen uptake. NMES studies determined its effect on cough capacity and pulmonary function by assessing FVC, FEV1, and PEFR. For FMS, expiratory reserved volume (ERV) and forced expiratory flow rate (FEF) were measured to evaluate FMS for assisting cough in people with tetraplegia. Both scTS studies evaluated its effectiveness for improving respiratory function by measuring MEP, MIP, ERV, and FEV1. These studies presented a large variety of outcome measures employed to evaluate respiratory function and cough using different noninvasive electrical stimulation modalities. The increased score of those outcome measures after the intervention was considered a sign of improvement for respiratory function and cough. The data indicate that functional outcome measures were greatly used for assessing respiratory function and cough; therefore, future studies are suggested to include kinetics, electromyographic, and electrophysiological measures that could provide additional evidence to validate these techniques.

### 3.6. Treatment Effects

Eleven out of nineteen studies have reported improved breathing and enhanced coughing ability after the administration of stimulation as an intervention for restoring respiratory and coughing function after SCI. In these studies, they have revealed the increased value of the following measures: VC, FVC, FEV1, PEFR, ERV, FEF, MEP, MIP, VT, total lung capacity, cough peak flow (CPF), MECP, etc. The prognosis was predicted based on the values recorded and monitored throughout the study including pre- and post-assessments.

Four out of fourteen FES studies exhibited increased abdominal muscle strength, endurance, and minute ventilation. Additionally, participants in these trials had less chest wall compliance, which resulted in creating abdominal pressure that triggered and induced effective cough. Two FES studies demonstrated increased expiratory pressure, expiratory flow, and expiratory volume during voluntary cough, which assisted in clearing airways and removed secretions, whereas one study demonstrated a rise in FVC, FEV1, and PEFR, showing improved pulmonary function. Additionally, four FES studies presented increased MEP, an elevation in gastric and esophageal cough pressures, and increased expiratory cough flow, causing abdominal muscle contraction, which enhanced and augmented coughing. Furthermore, two studies found an increase in VT, VC, VO2, and RPE, indicating improved aerobic fitness and enhanced respiratory function and hygiene. One FES study illustrated increased FVC that led to a decrease in respiratory complications. NMES studies recorded a rise in PEFR, FEV1, FVC, MEP, and MIP that provided abdominal support, which resulted in improved inspiratory capacity, cough capacity, and pulmonary function, with reduced pulmonary complications. Similarly, FMS reported an increase in ERV and FEV1, which helped in mobilizing bronchial secretions and restoring cough. The scTS measured increment of MIP, MEP, PEFR, FVC, FEV1, and spirometry measures demonstrated enhanced breathing and coughing ability, including improved episodes of dyspnea and hypophonia (Table 4). While the reviewed studies underreported adverse events, one study did note a small rise in arterial blood pressure during cough training sessions, which was associated with a modest autonomic dysreflexia response [63].

**Table 1:** Participant demographics and injury characteristics.

Author, Year	No. of Participants	Gender (M/F)	Mean Age (Years)	TSI (Years)	LOI, AIS
McBain et al. [64]; 2015	7	7/0	56	18	C4 AIS A, C4 AIS C; C5 AIS A, C5 AIS A; C6 AIS A; C7 AIS A, C7 AIS B
Philips et al. [34]; 1998	8	7/1	33	6.4	C6-C7; C8-T1; C8-T1; T4; T5; T8; T10; T12: complete SCI
Cheng et al. [37]; 2006	13	11/2	34.7	2.4	C4-C7: AIS A and AIS B
Gad et al. [48]; 2020	1	1/0	39	9	C5 AIS A
Duarte et al. [41]; 2021	4	3/1	33.1	-	Cervical SCI: AIS A
Lin et al. [42]; 1998	13	13/0	46.9	14.6	C4 AIS B, C4 AIS D; 6 C5 AIS A, 2 C5 AIS B; 2 C6 AIS A; C7 AIS A
Linder et al. [65]; 1993	8	8/0	38.4	12.3	C4-C5: complete SCI
Laghi et al. [66]; 2017	10	10/0	47.3	10.6	C5 AIS D; 2 C5-C6 AIS A; 3 C5-C7 AIS A; C5-C7 AIS B; C5-C7 AIS C; T4-T5 AIS C; T4-T6 AIS A
Gollee et al. [31]; 2007	4	3/1	36.7	1.8	C4, C4, C5, C6: complete SCI
Butler et al. [33]; 2011	11	6/5	45.5	9.2	C3-C4 AIS A; C4 AIS C; 2 C4-C5 AIS C; C4-C6 AIS A; C5-C6 AIS C; C6 AIS C; C7-T1 AIS A; T4 AIS A; 2 T6 AIS A
Jaeger et al. [67]; 1993	28	23/5	34.7	1.6	2 C4 AIS A; 4 C5 AIS A; 5 C5 AIS B, 2 C5 AIS C; 6 C6 AIS A; 5 C6 AIS B; 2 C7 AIS A; C7 AIS C, C7 AIS D
McBain et al. [63]; 2013	15	15/0	45.4	11.9	2 C4 AIS A; C4 AIS B, C4 AIS C; 2 C5 AIS A; C4-C5 AIS A; 2 C5-C6 AIS A; 2 C6-C7 AIS A; C6-C7 AIS B; C7 AIS C; T3 AIS A; T5 AIS A
Lee et al. [30]; 2008	1	11/0	65	15	C4 AIS A
Zupan et al. [68]; 1997	13	11/2	26.9	0.7	2 C4; 2 C5; 6 C6; 3 C7: 10 complete SCI, 3 incomplete SCI
Kumru et al. [57]; 2023	11	10/1	29.3	8.1	C3 AIS B; C4 AIS A, C4 AIS B, 2 C4 AIS C, C4 AIS D; C5 AIS B, C5 AIS C; C6 AIS A; C7 AIS B, C7 AIS C
Haviv et al. [69]; 2017	14	12/2	39.6	9.5	2 C4 AIS A; 2 C5 ASIA, 2 C5 AIS B; 2 C6AIS A; C6 AIS C; T2 AIS A; 4 T4 AIS A
Langbein et al. [29]; 2001	10	10/0	50.4	12.3	C4 AIS A, C4 AIS B, C4 AIS D; C6 AIS A, 3 C6 AIS B; C7 AIS D; T6 AIS A; T7 AIS A
McLachlan et al. [35]; 2013	12	11/1	38.5	16.3	C3 AIS C; 3 C4 AIS A, C4-C5 AIS A; C5 AIS A, C5-C6 AIS C; 2 C6 AIS A, 3 C6 AIS C
McCaughey et al. [32]; 2015	10	8/2	48.2	-	C3-C4 AIS A, C3-C4 AIS C; C4 AIS A, C4 AIS B, C4 AIS C; C5 AIS A, C5 AIS B, C5-C6 AIS A; C6-C7 AIS C; C7 A

(Male: M; female: F; time since injury: TSI; level of injury: LOI; American Spinal Cord Injury Association Impairment Scale: AIS).

**Table 2:** Study design, intervention delivered, and their delivery protocols.

Author	Study Design	Intervention	Intervention Delivery Protocols		
			Length of Delivery	Sessions/Week	Each Session Time
McBain et al. [64]	Experimental study	Functional electrical stimulation (FES)	-	-	-
Philips et al. [34]	-	FES	2–3 weeks	-	3–4 min
Cheng et al. [37]	Randomized controlled trial (RCT)	Neuromuscular electrical stimulation (NMES)	4 weeks	5	30 min
Gad et al. [48]	Case study	Transcutaneous electrical spinal cord stimulation (scTS)	2 weeks	5	60 min
Duarte et al. [41]	Retrospective case series	NMES	-	7 (twice each session)	20 min
Lin et al. [42]	Prospective before–after trial	Functional magnetic stimulation (FMS)	-	-	-
Linder et al. [65]	-	FES	-	-	30 min
Laghi et al. [66]	-	FES	-	-	1–2 min
Gollee et al. [31]	-	FES	-	-	-
Butler et al. [33]	-	FES	-	-	-
Jaeger et al. [67]	-	FES	-	-	-
McBain et al. [63]	Randomized crossover study	FES	6 weeks	5	-
Lee et al. [30]	-	FES	4 weeks	7	20–30 min
Zupan et al. [68]	-	FES	4 weeks	6 (twice each session)	20–30 min
Kumru et al. [57]	RCT	scTS	1 week	5	30 min
Haviv et al. [69]	-	FES	-	-	-
Langbein et al. [29]	Quasi experimental study design	FES	-	-	-
McLachlan et al. [35]	Longitudinal feasibility study	FES	3 weeks	7	60 min
McCaughey et al. [32]	Retrospective study	FES	8 weeks	5	20–40 min

**Table 3:** Stimulation parameters used for investigating respiratory function and cough after SCI.

Author	Stimulation Region	Frequency (Hz)	Pulse Width	Type of Pulse	Intensity (mA)
McBain et al. [64]	Abdominal muscle—posterolateral—and thoraco-abdominal wall	50 Hz	1 msec	Monophasic	~125 mA
Philips et al. [34]	Quadriceps, hamstrings gastrocnemius, and tibialis anterior muscle	35 Hz	300 msec	Biphasic	40–80 mA
Cheng et al. [37]	Pectoralis major and abdominal muscle	30 Hz	300 µsec	Biphasic	0–100 mA
Gad et al. [48]	C3–4, C5–6, or T1–2	30 Hz (carrier freq. 10 kHz)	1 msec	Biphasic	0–25 mA
Duarte et al. [41]	Midaxillary line at level of 6th, 7th, and 8th intercostal spaces	30 Hz	1 msec	-	60 mA
Lin et al. [42]	T10 to T11 spinous process	20 Hz	280 µsec	Biphasic	-
Linder et al. [65]	Abdominal wall	50 Hz	300 µsec	Biphasic	0–100 mA
Laghi et al. [66]	Rectus abdominis and external oblique muscle	50 Hz	250 µsec		100 mA
Gollee et al. [31]	Abdominal muscles	50 Hz	100–400 µsec	Monophasic	30–100 mA
Butler et al. [33]	Posterolateral abdominal muscles	50 Hz	200 µsec	-	50–150 mA
Jaeger et al. [67]	Midline on the abdomen	50 HZ	0.3 msec	-	-
McBain et al. [63]	Abdominal muscles—posterolateral	50 Hz	0.2 msec	-	50 mA
Lee et al. [30]	Anteriorly over abdomen	50 Hz	200 µsec	-	100 mA
Zupan et al. [68]	Abdominal muscles	50 Hz	0.3 msec	Monophasic	-
Kumru et al. [57]	C3-C4 and T9-T10	30 Hz (carrier freq. 10 kHz)	1 msec	Monophasic	~120 mA
Haviv et al. [69]	Abdominal muscles—above and under umbilicus	100 Hz	200 µsec	Monophasic	240 mA
Langbein et al. [29]	Abdominal muscles	-	250 µsec		~100 mA
McLachlan et al. [35]	Rectus abdominis and external oblique muscle	30 Hz	50 µsec	Biphasic	-
McCaughey et al. [32]	Rectus abdominis and external oblique muscle—bilaterally	30 Hz	100 µsec	Biphasic	30–150 mA

**Table 4:** Studies reporting electrode characteristics, outcome measures, and findings after introducing noninvasive stimulation modalities.

Study	Electrode Size	No. of Electrodes	Outcome Measures	Findings
McBain et al. [64]	10 × 18 cm^2^ 5 × 18 cm^2^	2 pairs	Expiratory volume, peak expiratory flow rate (PEFR), and gastric and esophageal pressure	Increased expiratory pressure, expiratory flow, and expiratory volume during voluntary cough, leading to effectively removing secretions.
Philips et al. [34]	-	-	Level of oxygen uptake (VO_2)_ and rate of perceived exertion (RPE)	Increase in VO_2_ and lowered RPE, which reduced fatigue and improved aerobic fitness.
Cheng et al. [37]	-	-	Vital capacity (VC), forced vital capacity (FVC), forced expiratory volume in 1 s (FEV1), and PEFR	Improved PEFR, FEV1, FVC, MEP, and maximal inspiratory pressure, with improved cough capacity and reduced pulmonary complications.
Gad et al. [48]	-	4 cathodes 2 anodes	Maximum inspiratory volume, PEFR, and FEV1	Improved breathing and coughing ability.
Duarte et al. [41]	-	2 channels	Tidal volume (VT) and VC	Reduced VT, pulmonary compliance, and increased inspiratory capacity.
Lin et al. [42]	Round 13.7 cm	-	Maximal expiratory pressure (MEP), expiratory reserve volume (ERV), and PEFR	Substantial increases in ERV and PEFR, mobilized secretions and restored cough.
Linder et al. [65]	-	-	ERV, lung volume, lung capacity, residual volume, and total functional residual capacity	Decreased VC, ERV, and total lung capacity, with increased residual volume and enhanced coughing ability.
Laghi et al. [66]		-	Esophagal and gastric pressure and airflow	Created abdominal pressure and lowered chest wall compliance, which induced cough.
Gollee et al. [31]	33 × 53 mm 50 mm round	4 channels	VT and cough peak flow (CPF)	Marked increased in VT, CPF, and minute ventilation, with improved cough and clearance of secretions.
Butler et al. [33]	2 pairs 4 × 18 cm	-	Total lung capacity, maximum expiratory pressure (MEP), inspiratory capacity (IC), VC, FVC, and FEV1	Increased expiratory pressure, PEFR, and lung volume and enhanced coughing.
Jaeger et al. [67]	Round 7.62 cm		PEFR	Increased in PEFR, which assisted in clearing airways.
McBain et al. [63]	10 × 18 cm	2 electrodes	Esophageal and gastric expiratory pressure and PEFR	Increased gastric and esophageal pressures and expiratory cough flow, significantly improving cough. *Adverse effect*: rise in arterial blood pressure associated with modest autonomic dysreflexic response.
Lee et al. [30]	3 × 8 cm	2 pairs	MEP, maximal expiratory cough pressure (MECP), PEFR, FEV1, and FVC	Increases in MEP, MECP, PEFR, FVC, and FEV1, which augmented cough and improved respiratory hygiene.
Zupan et al. [68]		2 pairs	FVC and FEV1	Increased abdominal muscle strength and endurance, improving cough.
Kumru et al. [57]	Round 2 cm 5 × 12 cm^2^	2 pairs	MIP, MEP, and spirometric measures	Significant improvement in episodes of breathlessness, dyspnea, hypophonia, MIP, MEP, and FVC.
Haviv et al. [69]	5 × 13 cm	4 electrodes	PEFR, FVC, and maximal voluntary ventilation (MVV)	Increased PEF, FVC, MVV, and produced abdominal muscle contraction that was triggered to induce cough.
Langbein et al. [29]	7.5 cm diameter	8 electrodes	FVC, FEV1, and PEFR	Improvement in FVC, FEV1, and PEFR, which could improve pulmonary function.
McLachlan et al. [35]	-	-	FVC, FEV1, PEFR, and MEP	Increase in FVC, FEV1, PEF, and MEP could lead to decrease respiratory complications.
McCaughey et al. [32]	33 × 53 mm	4 channels	VT and VC	Increased VC and VT could improve respiratory function.

### 3.7. Results of scTS Exploratory Trial

**Figure 4:**
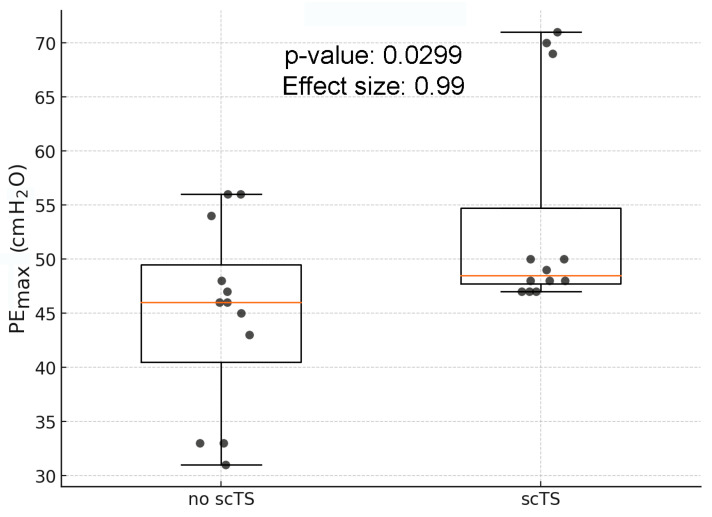
Maximum expiratory pressure (PEmax, cm H2O) at baseline (no scTS) and during the attempts accompanied by the scTS in two individuals with C4 AIS-B SCI. Note the significant increase (*p* = 0.029) in PEmax values in the presence of scTS and large effect size of such an increase (effect size = 0.99).

scTS significantly increased PEmax values from 44.83 ± 8.29 cm H_2_O assessed before stimulation to 53.67 ± 9.40 H_2_O recorded during the attempts accompanied by the scTS (Figure 4).

**Figure 5:**
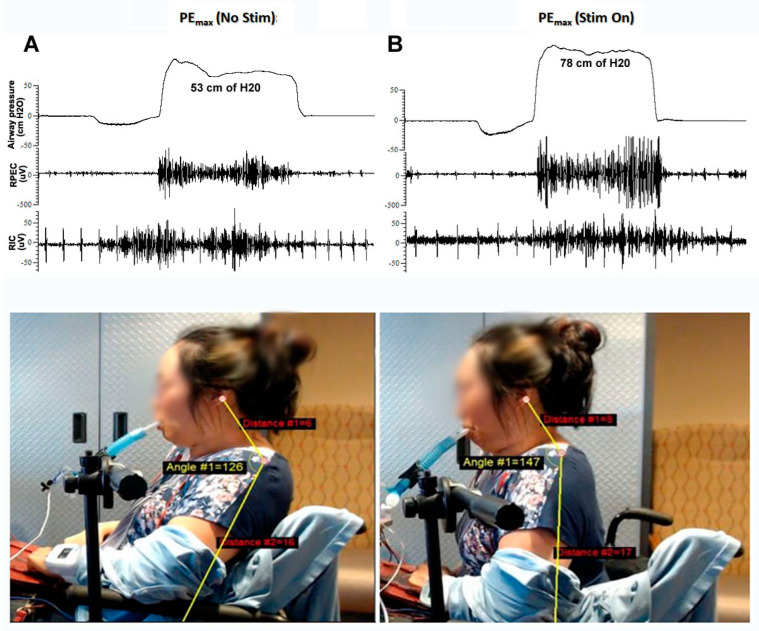
Airway pressure and sEMG from right pectoralis (RPEC) and right intercostals (RICs), and postural changes during PEmax efforts in an individual with C4 AIS-B SCI without (**A**) and during spinal cord transcutaneous stimulation (scTS) at T3 and T5 spinal levels (**B**). Note that scTS-induced activation of the spinal network results in increased maximum airway pressure (53 cm H_2_O vs. 78 cm H_2_O) in association with increased sEMG amplitude and active postural changes (neck–trunk angle 126° vs. 147°).

The effect size (Cohen’s d) for the comparison between the no scTS and scTS groups was 0.99, indicating a large effect size according to conventional benchmarks. This suggests a substantial difference between the two groups. This change was associated with increased sEMG amplitude and active postural changes (Figure 5).

## 4. Discussion

This study reviewed 19 articles highlighting the potential benefits of noninvasive electrical stimulation modalities in improving breathing, coughing, and related respiratory functions. Despite the critical importance of respiratory recovery as a component of rehabilitation following SCI, it remains underexplored, with pulmonary complications continuing to contribute significantly to morbidity and mortality [70]. Recent advancements in technology and innovation have positioned neuromodulation as a promising therapeutic approach. It has demonstrated improvements across various domains, including sensorimotor [71], upper limb [72], lower limb [73,74], trunk [19,75], and autonomic functions, such as respiratory function and cough [9,57]. The findings from this review suggest that pulmonary function can benefit from the application of electrical stimulation. This statement was confirmed by the result of the pilot trial using the most promising scTS parameters to activate respiratory motor networks. However, the lack of standardized stimulation parameters raises concerns about its efficacy and limits its translation into routine clinical practice.

This paper documented a wide range of stimulation characteristics and protocols, revealing significant gaps in the research focus on respiratory function. Notably, only 15.7% of the studies published in the last five years examined respiration, cough, and pulmonary functions. This highlights that research on respiratory recovery and associated impairments receives less priority compared to areas like locomotion [73,76] and upper limb recovery [71,77] in individuals with SCI. The importance of prioritizing respiratory function is underscored by the fact that mortality rates increase by 3% for every 1% decline in respiratory function [78], with pulmonary complications being the most common cause of death. Furthermore, the number of female participants was only 11.9%, which shows that men are mostly used in research studies. Given that females have lower proportion of SCI than males (1:2.3) [79], their participation in research is still very low. To ensure robust claims of efficacy and facilitate the effective clinical application of these stimulation modalities, it is essential to include both genders equitably in future studies. Addressing this imbalance will enable a more comprehensive understanding of the diverse characteristics of SCI populations and improve the generalizability of findings.

Many of the reviewed studies did not report AIS grades despite including individuals with varying SCI characteristics. The AIS score is an important tool for predicting neurological recovery and aids clinicians and researchers in setting treatment protocols and making informed decisions [80]. While recovery after SCI is influenced by the injury’s level, type, and severity, AIS characteristics can provide valuable insights into improvement prognosis [4]. Notably, individuals with incomplete SCI generally show a higher likelihood of recovery compared to those with complete SCI [21]. To improve research outcomes, future studies must categorize and specify injury characteristics, including AIS grades [75]. However, it is important to note that the AIS score has limitations in assessing respiratory or trunk muscle function. It assumes motor function aligns with sensory levels in the thoracic region, which may not accurately reflect respiratory impairment [81]. Additionally, the reviewed studies employed various stimulation configurations and protocols but often failed to specify which protocols were most effective for SCI characteristics. This lack of specificity makes it challenging to generalize the efficacy of these stimulation methods across all types of SCI. Future research should focus on investigating specific SCI subgroups, clearly reporting AIS grades and injury characteristics and systematically linking these factors to stimulation protocols. Such efforts are critical for developing tailored interventions that can achieve substantial results for diverse SCI populations.

The reviewed studies lacked detailed information regarding the sizes and shapes of stimulation electrodes, an oversight that may introduce variability in outcomes. Differences in electrode characteristics can influence stimulation intensity, potentially causing pain, discomfort, and inconsistent effects during transcutaneous stimulation [24]. Evidence suggests that electrode size impacts stimulation efficacy, with larger electrodes potentially altering the distribution and effectiveness of the current [82]. Moreover, while electrode placement was specified for FMS and scTS, it was not clearly described for FES and NMES. Even for FMS and scTS, it remains unclear whether single or dual stimulation sites were used. The Gerasimenko group demonstrated that dual-site stimulation, delivered in a rostro-caudal sequence—starting at a site closer to the head and progressing to a lower site—produced greater effects compared to stimulation at single sites [24]. Both animal and human studies have confirmed that multi-site scTS elicits more robust locomotor activity than single-site stimulation [83]. Clearly describing electrode properties, including size, shape, and placement, could enhance understanding and improve the practical application of these techniques. The transcutaneous approach, in particular, offers the flexibility to target multiple functions by repositioning electrodes to stimulate different spinal levels. This adaptability allows practitioners to identify the most effective sites for specific functions along the spine, providing a versatile noninvasive alternative to surgical interventions. This is especially beneficial for elderly individuals or growing children, for whom surgical options may carry higher risks or complications [71]. Future studies should prioritize the standardization and reporting of electrode characteristics and configurations to optimize the efficacy and safety of transcutaneous stimulation techniques.

The optimal timing, duration, and frequency of stimulation sessions necessary to achieve beneficial outcomes remains a subject of significant debate. The studies included in this review varied widely in their intervention durations and stimulation parameters. On average, reported interventions spanned 2–4 weeks, with 5–7 sessions per week lasting 30–60 min. However, many studies failed to provide details about these parameters, making it challenging to draw definitive conclusions about the optimal time required for effective stimulation. Previous research has shown that 20–30 min of continuous cervical scTS can modulate spinal and/or cortical networks controlling the upper limb muscles. Similarly, 30 min of lumbar scTS has been shown to regulate spinal reflex excitability [84]. Despite these findings, it remains unclear how the duration of stimulation impacts neural activity and long-term outcomes. The reviewed studies demonstrated considerable variability, with interventions lasting anywhere from a few weeks to several months. This inconsistency poses challenges for researchers and clinicians when attempting to implement these techniques in future studies. Many researchers suggest that extending the duration of interventions may lead to more durable therapeutic benefits [19,75]. Future research should aim to systematically investigate the effects of intervention duration, session length, and frequency on therapeutic outcomes. Identifying the optimal parameters—such as the ideal session length, number of weekly sessions, and total intervention duration—would provide valuable guidance for designing effective treatment protocols and planning future clinical trials. Standardizing these variables could also improve the comparability of studies and facilitate the translation of findings into clinical practice.

In this study, most FES applications utilized a stimulation frequency of 50 Hz to target respiratory muscles, suggesting that this frequency may be beneficial for FES. However, some studies employed frequencies as high as 100 Hz, hypothesizing that higher frequencies could enhance the therapeutic effects [35,65,69]. While frequencies of 50 Hz or higher have been shown to increase a muscle’s power-generating capacity, they may also contribute to muscle fatigue [32]. Consequently, lower stimulation frequencies, typically below 20 Hz, have been recommended to mitigate fatigue [85]. Frequencies in the range of 15–50 Hz have also been suggested to provide therapeutic benefits while minimizing fatigue [84]. For NMES and scTS, a consistent frequency of 30 Hz was used across all studies, indicating that 30 Hz may represent an optimal frequency for these modalities. Studies in humans and animals suggest that optimal frequencies for epidural stimulation vary based on the desired outcome, with 5–20 Hz for standing and 25–60 Hz for locomotion [50]. However, the most effective frequencies for transcutaneous stimulation remain uncertain, highlighting a need for further investigation. The reviewed studies applied stimulation intensities ranging from 25 to 240 mA, with the assumption that higher intensities would yield stronger therapeutic effects. Moderate muscle contractions were generally observed at around 40 mA, while stronger contractions were noted at 60–70 mA. Respiratory function measures, such as respiratory flow and pressure, improved with increasing stimulation intensity [86]. Optimal intensities for reducing pain and enhancing respiratory flow were reported to be between 60 and 80 mA [86]. The findings of this study align with these observations, as most studies reviewed employed intensities below 100 mA. However, there is no consensus in the literature regarding the most effective stimulation intensity for therapeutic outcomes [87]. The reviewed articles also varied in their use of pulse widths and intensities. While biphasic waveforms were commonly employed, monophasic waveforms were generally avoided in long-term applications due to the risk of tissue breakdown [37]. Biphasic waveforms, particularly symmetric configurations, were better tolerated by participants and allowed for higher stimulation intensities [76]. This likely explains their widespread adoption in studies utilizing stimulation as an intervention. To optimize the clinical application of these techniques, it is crucial to determine the ideal stimulation parameters, including frequency and intensity. Customizing the intervention to address the specific needs of each individual and adjusting stimulation configurations accordingly may maximize therapeutic benefits and improve clinical outcomes for individuals with SCI [57]. Further research is necessary to establish a consensus on these parameters and refine their use in practice.

Most of the reviewed studies reported improvements in respiratory function and cough, highlighting the positive effects of stimulation compared to conventional treatments. However, significant variability in outcomes and treatment effects was observed across the studies. These findings underscore the potential of noninvasive electrical stimulation modalities to transform neurorehabilitation, marking the dawn of a new era where unprecedented levels of improvement are achievable, even in the chronic stages of SCI. The emergence of neuromodulation techniques, particularly scTS, has introduced a clinically accessible approach that complements the existing state-of-the-art neuromodulation therapy [88]. Recent advancements in scTS have positioned it as a novel therapeutic strategy, showing promise for augmenting and restoring neurological functions, including respiratory recovery and cough [3,48,85]. While therapeutic benefits of scTS interventions have been demonstrated, the underlying neurophysiological mechanisms remain poorly understood and warrant further investigation [84]. Our study provides an overview of various stimulation modalities, including their characteristics and protocols, serving as a foundational resource for further exploration. Future research should address the variability in stimulation parameters, investigate neurophysiological mechanisms, and refine intervention protocols to maximize therapeutic benefits. By building on these findings, researchers and clinicians can advance the clinical application of these techniques and improve outcomes for individuals with SCI.

Noninvasive electrical stimulation modalities, including FES, NMES, and scTS, hold significant promise for clinical practice by offering innovative solutions to address respiratory impairments in individuals with SCI. Their noninvasive nature, ease of application, and adaptability make them particularly appealing for use in diverse clinical settings and patient populations. Current evidence suggests that these modalities can improve respiratory functions such as inspiratory and expiratory capacity, coughing, and secretion clearance, which are critical for preventing pulmonary complications [10,12,14]. The clinical application of these techniques has demonstrated that scTS can modulate spinal circuitries to enhance voluntary respiratory motor activity, even in chronic SCI patients [89]. This adaptability is further exemplified by the ability of scTS to target multiple spinal levels through strategic electrode placement, allowing clinicians to customize interventions for specific functional deficits [6,90]. Furthermore, NMES has shown promise in re-educating respiratory muscles and reducing spasticity, although its use requires careful parameter optimization to balance therapeutic effects and patient comfort [91].

Despite these advancements, the transition of these technologies into routine clinical practice remains limited by the lack of FDA-approved devices for home use and the variability in reported stimulation parameters. Home-based applications could revolutionize respiratory rehabilitation by enabling continuous cost-effective therapy, particularly for patients in the chronic phase of SCI [14]. Future research should focus on validating safe, user-friendly scTS devices suitable for home use, as well as establishing standardized stimulation protocols to maximize therapeutic efficacy [14,89]. Addressing these gaps will be pivotal in bridging the divide between experimental studies and real-world applications, ultimately improving patient outcomes.

### 4.1. Limitations

This study highlights the therapeutic potential of noninvasive electrical stimulation modalities for respiratory recovery in individuals with SCI. However, several limitations must be acknowledged, and they underscore key areas for future research as follows:

1. Heterogeneity in Study Designs and Protocols

The reviewed studies demonstrated significant variability in stimulation protocols, including differences in electrode configurations, stimulation parameters, and intervention durations. This heterogeneity complicates direct comparisons and limits the ability to draw definitive conclusions about optimal protocols. Standardized methodologies are essential to enhance comparability and facilitate clinical implementation.

2. Limited Sample Sizes and Demographic Representation

Many studies, including the reported exploratory trial, included small sample sizes and lacked diversity in participant demographics, particularly with underrepresentation of female participants. Given the physiological and anatomical differences between sexes, future research must aim for balanced representation to ensure findings are generalizable across diverse SCI populations.

3. Inconsistent Reporting of Injury Characteristics

Several studies failed to adequately document critical participant characteristics, such as the level and severity of SCI, using the American Spinal Injury Association (AIS) classifications. This omission hinders the ability to link stimulation protocols to specific injury profiles, limiting the applicability of findings to individualized care.

4. Short Intervention Periods and Lack of Long-Term Data

Most studies assessed outcomes over short durations, providing limited insight into the long-term sustainability of therapeutic effects. Future studies should include extended follow-up periods to evaluate whether improvements in respiratory function and cough are maintained over time.

5. Absence of Mechanistic Understanding

While scTS and other modalities have shown promise, the underlying neurophysiological mechanisms remain poorly understood. A deeper exploration of how stimulation parameters influence spinal and respiratory networks is critical for optimizing interventions and enhancing therapeutic outcomes.

6. Potential for Adverse Effects

Although adverse events were underreported, some studies noted issues such as discomfort, autonomic dysreflexia, and skin irritation at electrode sites. Future studies should systematically monitor and report adverse effects to ensure safety and refine stimulation protocols to minimize risks.

### 4.2. Future Directions

1. Development of Standardized Protocols

Future research should focus on developing standardized stimulation protocols, including optimized parameters such as frequency, intensity, pulse width, electrode configurations, and session durations. These standards will enable more consistent and reproducible outcomes across studies.

2. Personalized Stimulation Approaches

Tailoring stimulation protocols to the specific characteristics of the injury and the individual’s physiology will likely enhance therapeutic efficacy. Computational modeling and advanced imaging techniques could provide insights into the most effective stimulation sites and parameters for each patient.

3. Integration of Multimodal Interventions

Combining scTS with other rehabilitation techniques, such as respiratory training or pharmacological therapies, should be systematically explored. Research is needed to determine whether a simultaneous or sequential application of interventions produces superior outcomes.

4. Expansion of Study Populations

Future trials should include larger and more diverse populations, accounting for variables such as sex, age, and injury severity. This will improve the generalizability of findings and support the development of inclusive treatment strategies.

5. Mechanistic Studies

Investigating the neurophysiological mechanisms underlying scTS and other stimulation modalities will help elucidate their effects on respiratory networks. Techniques such as electrophysiology and functional imaging could provide valuable insights into how stimulation activates spinal and respiratory circuits.

6. Long-Term Efficacy Studies

Extended studies are needed to assess the durability of therapeutic effects and determine optimal maintenance strategies, including stimulation frequency and intensity adjustments over time. Understanding the long-term cost-effectiveness of interventions will also support their integration into clinical practice.

7. Minimizing Adverse Effects

Future research should prioritize the identification and mitigation of adverse effects associated with stimulation, including the use of innovative electrode materials and configurations to improve patient comfort and safety.

## 5. Conclusions

This study highlights the therapeutic potential of noninvasive electrical stimulation modalities, particularly scTS, for improving respiratory function in individuals with SCI. While the reviewed studies underscore the promise of these approaches in enhancing breathing, coughing, and related functions, significant gaps in standardization, reporting, and understanding remain.

Our findings emphasize the need for future research to address critical challenges, including the heterogeneity of study designs, the lack of standardized stimulation protocols, and the underrepresentation of diverse populations. In addition, multidisciplinary approaches, including pharmacological and physical therapy techniques, are important for future research investigating their complementary roles in respiratory rehabilitation. Standardizing parameters such as frequency, intensity, electrode configurations, and intervention durations will enhance the reproducibility of outcomes and facilitate the clinical translation of these techniques. Additionally, the development of personalized stimulation approaches tailored to the specific injury characteristics and physiology of individuals will likely optimize therapeutic efficacy.

To optimize stimulation protocols and address identified research gaps, we recommend future studies focus on standardizing stimulation parameters, including electrode size, shape, and placement, which are currently inconsistently reported and may influence outcomes. The establishment of optimal frequencies, intensities, and intervention durations is essential to maximize therapeutic benefits while minimizing side effects such as muscle fatigue. Moreover, systematic investigations should target specific SCI subgroups, categorized by level and completeness of injury, to enable tailored and effective interventions. Lastly, the integration of noninvasive electrical stimulation modalities into multidisciplinary rehabilitation programs, alongside pharmacological and physical therapies, could enhance overall patient outcomes. Such comprehensive research efforts will facilitate the development of replicable evidence-based protocols for widespread clinical application.

Although scTS has shown promising results in improving respiratory outcomes, including those demonstrated in our pilot data, the underlying neurophysiological mechanisms remain poorly understood. Mechanistic studies using advanced imaging and electrophysiological techniques are essential to elucidate how stimulation parameters influence spinal and respiratory networks. Furthermore, addressing the variability in intervention durations and evaluating long-term therapeutic benefits will provide valuable guidance for designing effective treatment protocols.

The variability in outcomes across studies highlights the need for robust methodological approaches, including the inclusion of larger, more diverse populations to ensure findings are generalizable. Expanding research efforts to include balanced representation across sexes, age groups, and injury severities is critical for developing inclusive and effective rehabilitative strategies. Finally, systematic monitoring and the mitigation of potential adverse effects will enhance the safety and acceptability of these interventions.

In conclusion, noninvasive electrical stimulation modalities represent a promising frontier in the rehabilitation of respiratory function for individuals with SCI. By addressing the identified gaps and refining intervention protocols, researchers and clinicians can advance the clinical application of these techniques, ultimately improving outcomes and quality of life for affected individuals.

## Data Availability

The data supporting the findings of this study are available upon request from the corresponding author. The protocol for the review was not prepared.

## Abbreviations

SCI	Spinal cord injury
ES	Electrical stimulation
FES	Functional electrical stimulation
NMES	Neuromuscular electrical stimulation
FMS	Functional magnetic stimulation
scTS	Spinal cord transcutaneous stimulation
FEV1	Forced expiratory volume in 1 s
PEFR	Peak expiratory flow rate (PEFR)
PEmax	Maximum expiratory pressure
PImax	Maximum inspiratory pressure
MECP	Maximum expiratory cough pressure
VO_2_	Level of oxygen uptake
RPE	Rate of perceived exertion
ERV	Expiratory reserve volume
FEF	Forced expiratory flow rate
FVC	Forced vital capacity
VC	Vital capacity
VT	Tidal volume
